# Miscellany

**Published:** 1906-09

**Authors:** 


### Miscellany.

NEW "STATE ASSOCIATION JOURNAL." — The *West Virginia Medical Journal*, published by the West Virginia State Medical Association, Vol. 1, No. 1, has been received. It is published by the Committee on Publication, Dr. S. L. Jepson, chairman, at Wheeling, W. Va. We find No. 1 quite interesting, and we add the fledgling to our exchange list with pleasure.

CAUSE AND EFFECT.—"My four-dollar and a half advertisement in your February number brought me *more than as many hundred* dollars, besides numerous letters of inquiry mentioning you.

"DR. J. W. KENNEY'S SANITARIUM,
"San Antonio, Texas."

[Dr. Kenney is now building a ten-room addition to his sanitarium. It is nearing completion.—ED.]

---

I can't afford to miss a single number of the "Red Back." I have never done so since I began practicing medicine.

Yours for success,

Richland Springs. A. D. NELSON.

---

There was once an old Woman Dr.
Whose patients' bad language oft shr.
Though she always would laugh
At their malice and chaugh,
And said that she cared not who knr.

—*International Journal of Therapy.*

---

TRI-STATE (ALABAMA, GEORGIA, TENNESSEE) MEDICAL SOCIETY.

The eighteenth annual meeting of the Tri-State Medical Society of Alabama, Georgia, and Tennessee will be held at Chattanooga, October 2-4, 1906. Reduced rates have been obtained from all points in Alabama, Georgia, Tennessee, Missisippi, Louisiana, and Florida, and an unusually large attendance is assured.

The preliminary program includes an excellent list of papers from leading medical men of the South. Strong pressure will be brought to bear to ultimately convert this organization into a branch of the A. M. A.—the Association of the Southeastern States —and recommendations will be made at this meeting.

Physicians desiring to read papers should send their titles at once to the secretary, Dr. Raymond Wallace, Chattanooga, Tenn.

---

DR. R. B. SELLERS, of Comanche, one of the best known, most popular and progressive of the younger M. D.'s, late assistant superintendent of the Southwestern Insane Asylum, San Antonio, has just completed a course at the New York Post-Graduate School, and reports for duty at home.

July 28, 1906.

*Dear Doctor Daniel:*

Here's to the "Red Back" and its staff. Many happy days. (Renewal for 1906-7.) Yours,

GEO. DOCK.

[University of Michigan, Ann Arbor.]

---

FRANKLIN, TEXAS, August 2, 1906.

*The "Red Back," Austin, Texas.*

DEAR DOCTOR: Find enclosed check for $2 for "Red Back." Can't do business without it. Fraternally,

J. C. HOLMAN.

---

DR. W. M. POWELL, for twenty-nine years practicing physician at Albany, Texas, and twenty-two years a subscriber to the "Red Back," has removed to Merkel, Taylor county, a rapidly-growing little city. He writes: "Let the good old 'Red Back' follow me up; I couldn't do without it."

---

THE "PACIFIC MEDICAL JOURNAL."—The first issue of this journal since the San Francisco disaster appears as a double number for May and June. It is devoted entirely to the medical aspects of that catastrophe, and is full of matter of absorbing interest. The entire plant of the *Journal* was destroyed by the fire, and with it went Dr. Winslow Anderson's large medical library, all the files of the *Journal,* and many manuscripts. The nucleus of a new library has been supplied by a number of the medical publishing houses, and the genius of the editor has revived his always interesting journal. Friends who have back numbers of the *Pacific Medical Journal* are asked to send them to Dr. Anderson, 1914 Pacific avenue, San Francisco, in order that the files may be reconstructed so far as possible.

---

A PROPOSED NATIONAL PUBLIC HEALTH SOCIETY.—Announcement is made of a meeting to be held in the Hudson Theater of this city on November 15 for the purpose of forming a national society for the preservation of public health. The movement is supported by a score of religious, medical, and scientific organizations, and has been endorsed by many persons of prominence. The object of the proposed society is stated to be to obtain and disseminate accurate information concerning practices and conditions of every kind that are dangerous to the public health and morals, to prevent quackery, criminal practices in the healing art, adulteration of drugs, and the sale of narcotics and alcohol under the guise of proprietary remedies.—*N. Y. Medical Record.*

